# Turning defense into damage: HIV-driven amyloidogenesis and neurotoxicity

**DOI:** 10.1128/mbio.03083-25

**Published:** 2026-04-21

**Authors:** Feng Gu, Badeia Saed, Mojgan H. Naghavi

**Affiliations:** 1 Department of Microbiology-Immunology, Northwestern University Feinberg School of Medicine 547641, Chicago, Illinois, USA; The Ohio State University, Columbus, Ohio, USA

**Keywords:** HIV, microglia, HIV-associated neurocognitive disorders, APP, C99, amyloidogenic processing, Gag, multivesicular bodies, neurotoxicity

## Abstract

With the continued spread of human immunodeficiency virus 1 (HIV-1) and its ability to enter and persist within the central nervous system (CNS), concerns have arisen regarding its impact on cognitive health. Indeed, during the early stages of the HIV pandemic, when effective treatments were unavailable, severe neurocognitive impairment was common. Although the widespread use of antiretroviral therapy (ART) has markedly reduced the severity, milder forms of HIV-associated neurocognitive disorders (HAND) remain prevalent. Similar to Alzheimer’s disease (AD), elevated amyloid-β (Aβ) accumulation has been observed both intracellularly and extracellularly in the brains of HIV-infected individuals, based on autopsy studies. Aβ is generated through the amyloidogenic processing of amyloid precursor protein (APP), which is abundantly expressed in the brain. While the APP’s role in AD pathogenesis has been well established, its broader physiological functions, particularly in the context of viral infections such as HIV-1, remain poorly understood. In the CNS, microglia are crucial for maintaining brain homeostasis and defending against viral infections. HIV-1, however, targets microglia, disrupting their antiviral capacity and contributing to neurotoxicity through multiple mechanisms, such as the release of viral proteins and host-derived neurotoxic factors including proinflammatory cytokines and Aβ. Moreover, HIV-infected microglia can influence neighboring cells such as astrocytes and neurons, further amplifying neurodegenerative processes. This review will focus on recent advances in understanding the antiviral role of APP and its processing during HIV-1 infection, highlighting how APP-mediated defense mechanisms intersect with neurotoxic pathways and the intercellular regulatory networks that link APP to HAND.

## INTRODUCTION

## HIV-ASSOCIATED NEUROCOGNITIVE DISORDERS

Since the beginning of the HIV pandemic, approximately 91 million people have been infected, and over 40 million are living with the virus worldwide in 2024 (1). Despite the widespread availability of ART that suppresses viral loads to undetectable levels, 40%-50% of people living with HIV continue to experience HAND of varying severity (2–4). Neurological impairments have been reported in HIV-1 patients, though whether the virus was the direct cause was debated in the early stages. However, by 1987, Grant et al. provided evidence linking HIV-1 infection to neurocognitive impairment (5). Soon after, diagnostic guidelines were developed, and the most recent amended criteria, published in 2007, formally introduced the term HAND and defined it in three categories: asymptomatic neurocognitive impairment (ANI), mild neurocognitive disorder (MND), and HIV-associated dementia (HAD) (6). However, a recent viewpoint article argued the current criteria may cause over 20% false positives and should be revised in the modern ART era, where treatment access and efficacy have significantly improved (7). Despite advances in diagnosis, understanding how HIV-1 gains access to and persists within the brain remains a challenge in elucidating HAND pathogenesis.

Once infection is established, HIV-1 can enter the CNS through the “Trojan Horse” mechanism, in which infected monocytes and T cells cross the blood-brain barrier (BBB) (8–10). The virus is also thought to be capable of directly penetrating the brain by increasing BBB endothelial permeability, caused by viral factors, such as the viral trans-activator of transcription (Tat) and glycoprotein 120 (gp120) (11–14). Consequently, HIV-1 RNA can be detected in the CNS as early as 8 days after the estimated time of infection (15). With two possible routes to enter the CNS, HIV-1 primarily infects and establishes latency in microglia in the brain, with minimal infection of astrocytes and virtually no infection of neurons (16). Upon reactivation from latency, viral replication shifts microglia from a resting to an active state, resulting in the release of viral proteins, such as Tat, gp120, and negative regulatory factor (Nef), all of which are reported to directly damage neurons (17). Additionally, infected microglia secrete proinflammatory factors, including tumor necrosis factor-alpha (TNF-α), interleukin-1 beta (IL-1β), interleukin-6 (IL-6), as well as Aβ, contributing to further HIV-1 reactivation and indirect neurotoxicity (16, 18).

**Fig 1:**
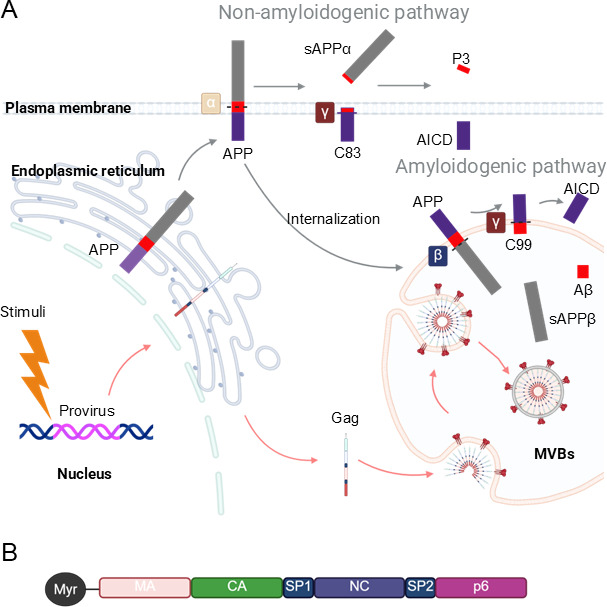
Intersecting pathways of APP processing and HIV-1 Gag trafficking in microglia. (**A**) Following synthesis in the endoplasmic reticulum, APP is transported to plasma membrane, where it undergoes non-amyloidogenic processing. In this pathway, α-secretase cleaves APP to release N-terminal soluble APPα (sAPPα) and generate plasma membrane-bound C-terminal fragment C83. C83 is subsequently cleaved by γ-secretase to produce extracellular P3 peptides and APP intracellular domain (AICD). APP that is not processed at the plasma membrane is rapidly internalized and transported to late endosomes, where MVBs form. Within this compartment, APP is processed by the amyloidogenic pathway: β-secretase cleaves APP to generate luminal N-terminal soluble APPβ (sAPPβ) and the membrane-tethered C99 fragment, which is further processed by γ-secretase to produce luminal Aβ and AICD released into the cytosol. Upon cellular activation, integrated HIV-1 provirus is expressed, and the viral structural polyprotein, Gag, is synthesized and trafficked to MVBs for assembly. Premature viral particles are released into the lumen of MVBs. (**B**) HIV-1 Gag is a 55-kDa polyprotein composed of matrix (MA), capsid (CA), spacer peptide 1 (SP1), nucleocapsid (NC), spacer peptide 2 (SP2), and p6 domain. The N-terminal glycine of the MA domain is covalently modified with a myristoyl (Myr) group, catalyzed by the host enzyme N-myristoyltransferase. This N-myristoylation is essential for targeting Gag to the plasma membrane or MVB membrane. This figure was created with BioRender.com.

Interestingly, Aβ is a well-established hallmark of AD and is generated through sequential proteolytic cleavage of APP via the amyloidogenic pathway within endocytic compartments (Fig. 1A). Extracellular accumulation of secreted Aβ, particularly the Aβ42 isoform, contributes to neuronal dysfunction and neurodegeneration (19). Clinical studies have indicated that Aβ deposition is accelerated in the brains of people living with HIV (20, 21). In line with this, several HIV proteins have been found to be involved in Aβ accumulation. For example, Tat and gp120 have been shown to disrupt APP processing and promote Aβ42 deposition (22–24). Tat can also bind directly to Aβ, forming extracellular Tat-Aβ complexes that exhibit greater neurotoxicity than Aβ alone (25). In addition, an *in vitro* study suggested that Tat impairs microglial clearance of Aβ, thereby exacerbating Aβ accumulation and neuronal injury (26). Despite these advances, the molecular mechanisms underlying APP function, specifically, why it is processed into neurotoxic Aβ during HIV-1 infection, remain poorly understood.

## ROLES OF MICROGLIA DURING HIV-1 INFECTION AND HAND OCCURRENCE

Microglia, originating from erythro-myeloid progenitors in the yolk sac and subsequently migrating into the developing brain parenchyma, were clearly defined in the early 20th century by the Spanish neuroscientist Pío Del Río Hortega, who described their capacity to migrate to sites of injury and clear cellular debris (27, 28). Although microglia make up only about 10% of CNS cells and 20% of glial cells, they play crucial roles in brain development, homeostasis, and disease (29, 30). As the primary resident immune cells of the CNS, microglia play key roles in antiviral defense (31). Neurotropic viruses, including retroviruses, coronaviruses, herpes simplex virus type 1 (HSV-1), Zika virus, and Japanese encephalitis virus (JEV), can infect and manipulate microglia, triggering neuroinflammation and, in severe cases, neurodegeneration (32). Neurons and astrocytes, which constitute the majority of CNS cells, are generally non-permissive to productive HIV-1 infection in large part because they do not express the necessary surface receptors for efficient viral entry (33, 34). Although HIV can enter astrocytes through other receptor-independent pathways, and it is still unknown if they constitute a productive cellular reservoir (34, 35), changes in astrocyte gene expression caused by infection can also contribute to HAND, as described in more detail below. In contrast, microglia, although expressing lower levels of cluster of differentiation 4 (CD4) than T cells, remain susceptible to infection by macrophage-tropic (M-tropic) HIV-1 strains that utilize C-C chemokine receptor type 5 (CCR5) as a co-receptor (36). Microglia express higher levels of functional CCR5 than C-X-C chemokine receptor type 4 (CXCR4), an alternative co-receptor, and blocking CCR5 with neutralizing antibodies strongly inhibits infection (37). These features characterize microglia as an ideal target cell for HIV-1 and a major viral reservoir in the CNS, where they can persist even under ART due in part to low penetrance of antivirals into the brain (38, 39).

Upon infection of immune cells ranging from T cells to macrophages or microglia, HIV-1 integrates its genetic material into the host genome to form a provirus (39, 40). Once activated, the provirus initiates viral gene expression (41). In microglia, current models of HIV-1 latency establishment are largely based on *in vitro* studies, involving suppression of proviral transcription by recruiting host factors to HIV-1 promoter long terminal repeat (LTR) and reduction of inflammatory responses (16, 38). For example, the nuclear receptor related 1 (Nurr1), a member of the nerve growth factor-induced β subfamily of orphan nuclear receptors, has been characterized as an anti-inflammatory and neuroprotective factor implicated in neurodegeneration (42, 43). In HIV-1 latent microglia cell models, Nurr1 binds the HIV-1 LTR and recruits the corepressor element 1-silencing transcription factor (CoREST) complex to silence proviral transcription (44). Similarly, the glucocorticoid receptor (GR) is a constitutively expressed transcription regulator, and its activation in microglia protects brain from innate immune responses (45, 46). An *in vitro* study showed that glucocorticoid-mediated activation of GR suppresses spontaneous HIV-1 reactivation driven by autocrine of TNF-α in microglia (47). Notably, Nurr1 recruitment and GR activation also inhibit microglial inflammation by repressing NF-κB signaling and TNF-α secretion, respectively (44, 47).

HIV-1 reactivation in microglia is strongly dependent on host transcription regulators, particularly NF-κB (16). Proinflammatory stimuli, such as TNF-α activate NF-κB, which binds the LTR and drives viral transcription and protein expression (16, 47). A recent study of HIV-1 positive human brain samples showed that, even under ART, HIV DNA persists in microglia and a small subset of astrocytes (48). Approximately half of HIV DNA-positive cells express viral mRNA, and about one-third produce viral proteins, including the polyprotein group-specific antigen (Gag) and its cleaved p24 capsid protein, gp120, and Tat (48). Notably, viral proteins, such as p24, gp120, Nef, and Tat, are also detected in neighboring uninfected cells, indicating ongoing secretion and uptake of viral proteins within the brain microenvironment, which may contribute to chronic inflammation and promote HIV-1 reactivation (48).

Among the expressed viral proteins, the structural polyprotein Gag, a 55kDa precursor composed of matrix (MA), capsid (CA), nucleocapsid (NC), p6 domain, and two spacer peptides (SP1 and SP2), plays a central role in virion assembly and budding (Fig. 1B) (49). In T cells, the MA domain targets Gag by interacting with phosphatidylinositol 4,5-bisphosphate (PI(4, 5)P2) to the plasma membrane, where the CA domain drives Gag multimerization (49). The NC domain recruits the viral RNA genome into assembling virions, while the p6 domain mediates virus budding by recruiting the host endosomal sorting complex required for transport (ESCRT) machinery (49). This machinery includes the tumor susceptibility gene 101 (TSG101), the key component of ESCRT-I, and the vacuolar protein sorting 4 (VPS4), which catalyzes membrane scission leading to virion release (50, 51). The p6 domain directly binds TSG101 through its Proline-Threonine-Alanine-Proline (PTAP) motif, thereby recruiting ESCRT-III and VPS4 to complete releasing (50, 51).

However, in “professional” phagocytes, such as macrophages and microglia, HIV-1 assembly and budding primarily occur within multivesicular body (MVB)-like compartments, also referred to as virus-containing compartments (VCCs) (52–56). While retaining key features and markers of intracellular MVBs, VCCs are also thought to be connected via thin channels to the cell surface as a form of extreme invagination of the plasma membrane (57). Multiple viral and host factors have been found involved in regulating Gag targeting MVBs in phagocytes (54, 55, 58–61). Among them, APP is highly expressed in microglia and macrophages (62), and as discussed below, its complex sorting and processing through secretory pathways intersects with HIV-1 replication at MVBs/VCCs in ways that both impact virus replication and the production of toxic amyloids (55). In addition, one of the crucial functions of microglia in maintaining CNS homeostasis is the clearance of Aβ through phagocytosis. However, HIV-1 infection impairs microglial Aβ clearance, primarily due to the disruptive effects of the viral protein Tat, which interferes with signaling pathways regulating phagocytosis and alters the expression of phagocytic receptors. This impairment leads to extracellular Aβ accumulation and contributes to neurodegeneration (63). Overall, microglia are essential for maintaining brain homeostasis and serve as the first line of defense against HIV-1 infection. APP plays a key role in both restricting virus replication and influencing the production of neurotoxic products that contribute to HAND.

## APP AMYLOIDOGENIC PROCESSING IN MICROGLIA DURING HIV-1 INFECTION

APP was first purified and characterized in association with AD by George G. Glenner and Caine W. Wong in 1984 (64). Subsequent research rapidly expanded our understanding of APP, leading to the identification of alternative transcripts encoding APP695, APP751, and APP770 (65), the discovery of familial AD-linked mutations in APP (66, 67), the characterization of α-, β-, and γ-secretase cleavage pathways (68), the identification of APP homologs (69), and the recognition of endosomal trafficking as a key determinant of amyloid generation (19, 70). APP is widely expressed in many tissues; but in the brain, the shortest isoform APP695 predominates in neurons, whereas glial cells, including microglia and astrocytes, express higher levels of APP751 and APP770 (71, 72). APP performs diverse functions, primarily acting as cell surface receptors or ligands in pathways that regulate CNS development; importantly, its aberrant proteolytic processing into neurotoxic Aβ contributes to AD pathogenesis (73).

APP is primarily processed via two well-characterized pathways: the non-amyloidogenic and amyloidogenic pathways (Fig. 1A) (74). The sorting and subcellular localization of APP and its secretases determines the outcome of APP processing (74). As a type I transmembrane protein, APP is initially synthesized in the endoplasmic reticulum (ER) and then transported to the plasma membrane via the secretory pathway (74). Once at the plasma membrane, APP is primarily processed through the non-amyloidogenic pathway, where α-secretase first cleaves APP to generate the membrane-bound C83, also termed alpha-C-terminal fragment (α-CTF) and ectodomain soluble APP alpha (sAPPα); subsequently, C83 is rapidly cleaved by γ-secretase to produce the extracellular P3 peptide and the APP intracellular domain (AICD) (75).

Within minutes, any remaining non-cleaved APP is rapidly internalized from plasma membrane into endosomes, a process driven by its carboxyl-terminal sorting signal, the asparagine-proline-threonine-tyrosine (N-P-T-Y) motif (76, 77). This motif recruits multiple host adaptor proteins to mediate clathrin-dependent endocytosis (19). The internalized APP is cleaved by β-secretase (primarily beta-site APP cleaving enzyme 1 [BACE1]) within endosomes, where the acidic environment optimizes its activity, resulting in the generation of the membrane-tethered C99 fragment, also termed beta-C-terminal fragment (β-CTF) (78, 79). C99 is subsequently recognized and cleaved by γ-secretase within endosomes, generating neurotoxic Aβ peptides and AICD (19). Numerous pathogenic mutations that promote amyloidogenic processing have been identified in APP (80). For example, the Swedish mutations at codon K670N and M671L (APP 770 numbering) cause early-onset AD by enhancing β-secretase cleavage of APP, thereby increasing the production of neurotoxic Aβ peptides (67, 81). Once generated in endosomes, Aβ peptides are trafficked to MVBs and released in exosomes upon MVB-plasma membrane fusion (82).

These findings underscore the critical role of endosomes in the amyloidogenic processing of APP. As such, modulating endocytosis or endosomal activity can markedly impact Aβ generation. Indeed, blocking endocytosis reduces APP internalization and consequently decreases Aβ production (83), whereas alkalinization of endosomal pH impairs amyloidogenic APP processing and Aβ generation (84). Conversely, enhanced endocytosis accelerates amyloidogenic APP processing and Aβ production (85). HIV-1, a potent cellular disruptor, exploits late endosomes in microglia to facilitate viral production, thereby perturbing endosomal function and potentially impacting amyloidogenic APP processing. Indeed, a recent study revealed that HIV-1 Gag enhances amyloidogenic processing of APP C99, elevating Aβ generation in infected microglia (55). Gag binding to TSG101 is essential for recruiting ESCRT machinery to mediate viral assembly and budding at the plasma membrane in T cells (51). A follow-up study showed that, in microglia, disrupting the Gag-TSG101 interaction prevents Gag trafficking to late endosomes and protects APP C99 from amyloidogenic processing (56). These findings highlight the key role of endosomal trafficking in amyloidogenic APP processing and Aβ production and reveal how HIV-1 hijacks this pathway in microglia, coupling viral replication to increased neurotoxic Aβ generation.

## ROLES OF APP AND CLEAVED PRODUCTS IN HIV-1 INFECTION

While APP metabolism and Aβ effects are well characterized in neurodegeneration, its roles beyond AD remain less well understood. A review by Dawkins et al. proposed that APP has broad trophic function, including regulating neural stem cell development, neuronal survival, and neurite outgrowth (86). Recent emerging evidence links APP to viral infections (62, 87–89). For example, APP can act as a proviral factor for SARS-CoV-2 by promoting viral entry through interaction with the viral spike protein (89). In contrast, during Zika virus infection, APP is stabilized and restricts viral replication in neural stem cells by binding the viral E protein, a primary viral protein for attachment to host cells (88). HSV-1 infection enhances APP processing into multiple neurotoxic fragments of different lengths in rat and human neuronal cells, although its impact on HSV-1 infection remains unclear (87). In the context of HIV-1, APP functions as an antiviral factor in microglia and macrophages. It interacts with HIV-1 Gag in lipid rafts, impeding virion release. However, Gag counteracts this restriction by promoting APP cleavage toward amyloidogenic pathway, resulting in increased production of neurotoxic Aβ42 (62). As Aβ42 is generated directly through C99 cleavage, and HIV-1 transgenic rat models have shown significant accumulation of C99 in the brain (90), subsequent studies investigated the role of C99 during HIV-1 infection and found that either C99 overexpression or inhibition of its cleavage with γ-secretase inhibitors suppresses virion release, suggesting that C99 exerts antiviral function (55). Further analysis revealed that C99 localizes to late endosomes and MVBs, which are essential for Gag assembly, where it blocks Gag entry (55). To overcome this host antiviral mechanism, Gag promotes C99 ubiquitination at multiple sites, facilitating its cleavage and thereby enabling Gag trafficking into MVBs (55). In contrast, the non-amyloidogenic fragment C83 neither inhibits virion production nor undergoes Gag-induced ubiquitination (55). Surprisingly, overexpression of C83 actually enhances HIV-1 production through an as-yet unknown mechanism (55). Given that C83 predominantly resides at the cell surface whereas C99 localizes to late endosomes, this highlights the critical role of subcellular trafficking in coordinating C99 processing and Gag assembly. It also suggests that host factors governing intracellular trafficking are key regulators of this interplay. Indeed, knockdown of TSG101 was found to block Gag trafficking into MVBs and restore C99 cleavage in microglia (56). VPS4A is a well-established late-acting ESCRT ATPase essential for virion particle budding at the plasma membrane in T cells (51). In microglia, VPS4A also interacts with and stabilizes C99, a factor that negatively affects Gag trafficking into MVBs and reduces HIV-1 production (56). It appears that as HIV-1 co-opts VPS4A for its own replication, it also disrupts the C99-VPS4A interaction, which in turn helps the overall process of accelerating C99 amyloidogenic processing into Aβ during infection (56). These findings demonstrate that while APP-derived C99 restricts HIV-1 replication, the virus hijacks several regulatory factors in this pathway to promote C99 processing into Aβ, converting an antiviral defense into a neurotoxic outcome. Due to the limited efficacy of ART against latent CNS reservoirs, the continuous spread and infection in the brain mediated by viral countermeasures may spread out of the brain and into peripheral organs (91).

## APP-ASSOCIATED INTERCELLULAR NEUROTOXICITY IN THE CONTEXT OF HIV-1 INFECTION

**Fig 2:**
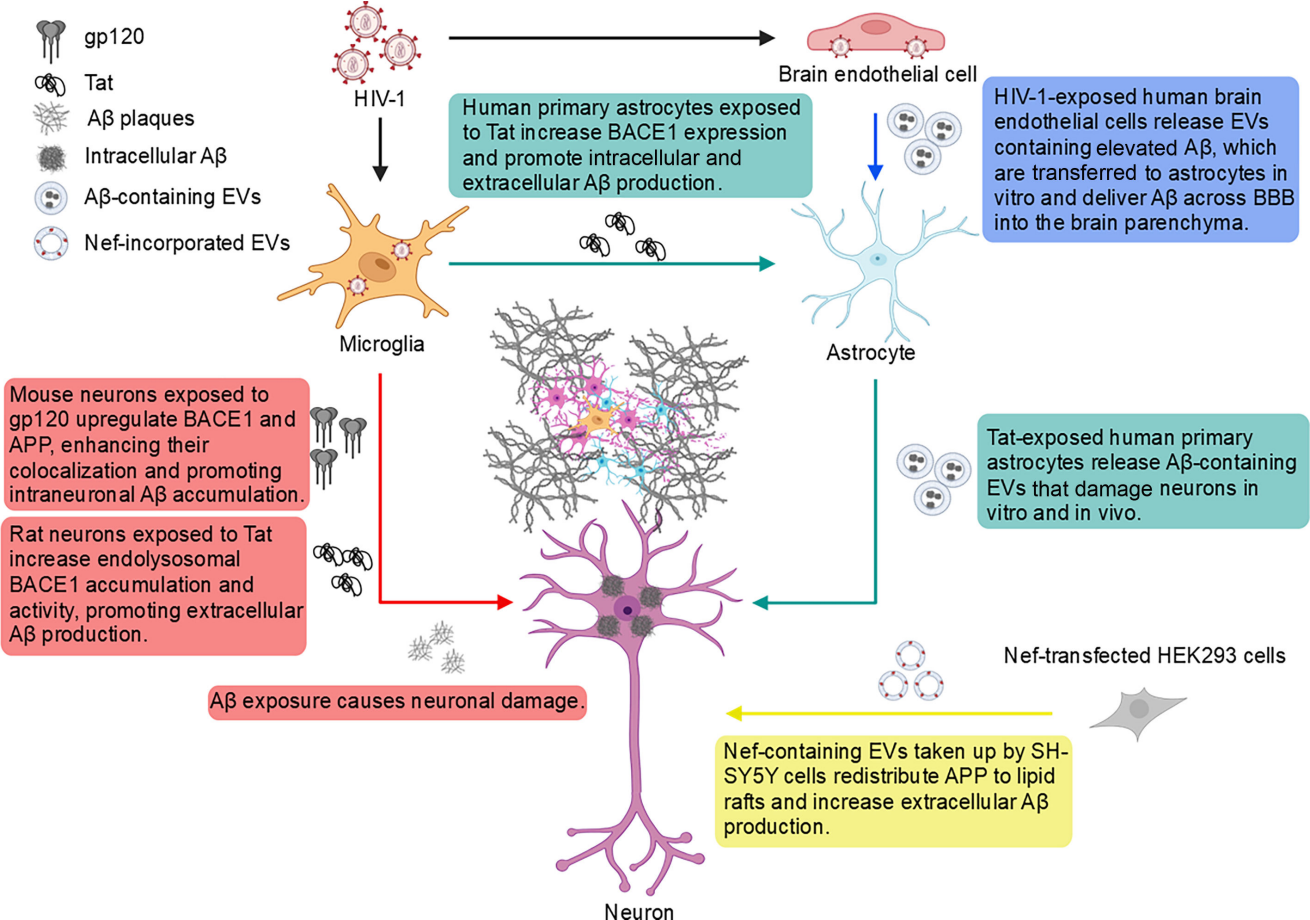
APP-associated intercellular communication in the CNS during HIV-1 infection. Microglia serve as a major reservoir for HIV-1 within the CNS. Aβ released from infected microglia contributes to neurotoxicity (red arrow). Viral proteins, such as Tat and gp120, released from infected microglia promote neuronal Aβ accumulation (red arrow). Additionally, Tat can induce Aβ production in astrocytes, which in turn may cause neuronal damage by releasing EVs enriched in Aβ42, which are internalized by neurons (green arrows). HIV-1 may also infect endothelial cells of the blood-brain barrier (BBB), potentially facilitating the transfer of Aβ to astrocytes (blue arrow). Furthermore, EVs carrying HIV-1 Nef protein that are taken up by neurons may disrupt APP trafficking and enhance Aβ42 production (yellow arrow). All these intercellular interactions contribute to a toxic intracellular and extracellular environment for neurons. This figure was created with BioRender.com.

Despite disrupting APP’s antiviral activity by accelerating its amyloidogenic processing in infected microglia, intercellular communication among CNS cells further contributes to neuronal damage during HIV-1 infection by altering APP metabolism in other cell types, particularly in astrocytes and neurons. Recent studies have uncovered a complex interplay between APP metabolism and HIV-induced intercellular alterations, highlighting how Aβ both regulates and is regulated by factors released from HIV-infected cells, thereby promoting neurodegeneration through direct and indirect mechanisms (Fig. 2) (24, 92–97). Neurons, although not directly susceptible to HIV infection, are profoundly affected by viral proteins and soluble factors, notably Aβ, secreted from infected cells, especially microglia (16, 62). Indeed, infected microglia release viral proteins, such as gp120, Tat, and Nef, which mediate Aβ generation across cells (24, 92). For example, HIV gp120 was found to increase both BACE1 and APP expression, leading to intracellular Aβ accumulation in mouse neurons (24), while exposure to Tat disrupts neuronal endosomal function and promotes Aβ deposition (92). HIV Nef contributes to viral pathogenesis by enhancing HIV replication and evading host immune restriction (98). Several studies have reported high levels of Nef-containing extracellular vesicles (EVs) in the blood of HIV-1 infected patients, correlating with viral pathogenesis (99, 100). *In vitro*, Nef carried by EVs is taken up by human neuroblastoma cells, redistributing APP to lipid rafts and increasing Aβ42 production (95). In mice, treatment with Nef-containing EVs elevated APP levels in brain tissues (95). Similarly, secreted factors from HIV-infected monocyte-derived macrophages (MDMs) enhance extracellular Aβ42 oligomer release from primary rat neurons by upregulating BACE1 expression via N-methyl-D-aspartate receptor (NMDAR) activation (94). In addition to neurons, astrocytes, although inefficiently infected by HIV-1, can be activated by viral proteins and transmit neurotoxic effects to neurons (96, 97). Tat exposure increases intracellular Aβ42 production in human primary astrocytes by upregulating BACE1 expression mediated by hypoxia-inducible factor one subunit alpha (HIF1α) (96). A recent follow-up study further demonstrated that the Tat-stimulated astrocytes release Aβ-enriched EVs, which are internalized by neurons and induce synaptodendritic injury both *in vivo* and *in vitro* (97). Endothelial cells of the BBB may also contribute to intercellular Aβ transfer; limited *in vitro* studies suggest HIV-1 induces Aβ accumulation in brain endothelial cells and promotes EV-mediated Aβ transport across the BBB to adjacent astrocytes (93, 101), but the *in vivo* consequences remain unclear. Altogether, these findings emphasize the intricate nature of intercellular communication in the HIV-1-infected brain. While significant progress has been made in elucidating how HIV-1 alters APP metabolism across different CNS cell types, further studies are needed to fully understand the complex mechanisms of cell-cell interactions that contribute to HAND.

## CONCLUSION

HIV-1 infection profoundly perturbs APP metabolism within the CNS. Although APP and its intermediate fragment, C99, possess intrinsic antiviral functions, HIV-1 exploits this pathway by accelerating amyloidogenic processing in microglia, resulting in excessive Aβ production. The release of Aβ, along with viral proteins and other host pathogenic factors from infected microglia and stimulated CNS cells, amplifies neuronal damage both directly, through neurotoxic effects, and indirectly, by disrupting intercellular communication among microglia, astrocytes, and neurons. Collectively, these findings position APP as both a target and mediator of HIV-1-induced neurotoxicity, underscoring its pivotal role in the progression of HAND.
